# The application of point source electroporation and chemotherapy for the treatment of glioma: a randomized controlled rat study

**DOI:** 10.1038/s41598-020-59152-7

**Published:** 2020-02-07

**Authors:** Shirley Sharabi, David Guez, Dianne Daniels, Itzik Cooper, Dana Atrakchi, Sigal Liraz-Zaltsman, David Last, Yael Mardor

**Affiliations:** 1 The Advanced Technology Center, Sheba Medical Center, Ramat-Gan, 52621 Israel; 2 Sackler Faculty of Medicine, Tel-Aviv University, Tel-Aviv, Israel; 3 The Joseph Sagol Neuroscience Center, Sheba Medical Center, Tel Hashomer, Ramat Gan, Israel; 4 Interdisciplinary Center Herzliya, Herzliya, Israel; 5 Gand Faculty of Health Profession, Ono Academic College, Kiryat Ono, Israel; 6 Department of Pharmacology, Institute for Drug Research, Hebrew University, Jerusalem, Israel

**Keywords:** Preclinical research, Cancer therapy

## Abstract

The prognosis of Glioblastoma Multiforme patients is poor despite aggressive therapy. Reasons include poor chemotherapy penetration across the blood-brain barrier and tumor infiltration into surrounding tissues. Here we studied the effects of combined point-source electroporation (EP) and systemic chemotherapy in glioma-bearing rats. 128 rats were studied. Treatment groups were administered systemic Cisplatin/Methotrexate before EP (either 90 or 180 pulses). Control groups were treated by EP, chemotherapy, or no treatment. Tumor volumes were determined by MRI. Tumors growth rates of the EP + Methotrexate group (1.02 ± 0.77) were significantly lower (p < 0.01) than the control (5.2 ± 1.0) 1-week post treatment. No significant difference was found compared to Methotrexate (1.7 ± 0.5). Objective response rates (ORR) were 40% and 57% for the Methotrexate and EP + Methotrexate groups respectively. Tumor growth rates and ORR of the EP + Cisplatin groups (90 pulses 0.98 ± 0.2, 57%, 180 pulses 1.2 ± 0.1, 33%) were significantly smaller than the control (6.4 ± 1.0, p < 0.01, p < 0.02, 0%) and Cisplatin (3.9 ± 1.0, p < 0.04, p < 0.05, 13%) groups. No significant differences were found between the control groups. Increased survival was found in the EP + Cisplatin group, Χ^2^ = 7.54, p < 0.006 (Log Rank). Point-source EP with systemic chemotherapy is a rapid, minimal-invasive treatment that was found to induce significant antineoplastic effects in a rat glioma model.

## Introduction

Despite aggressive therapy, the mean survival of Glioblastoma multiforme (GBM) the most common and aggressive primary tumor of the central nervous system (CNS) is only ~14 months. The standard of care since 2005 includes surgery, chemoradiation with Temozolomide and then adjuvant Temozolomide^1^.

Several factors contribute to the poor prognosis including limited efficiency of most therapeutic agents due to poor blood-brain barrier (BBB) penetration^2,3^ and high resistance to apoptotic stimuli. On top of this, infiltration of tumor cells into the surrounding parenchyma induced tumor recurrence which is expected in most patients within 6–9 months of initial treatment protocol^4^. 90% of recurrence is at the margin of surgical resection, within the macroscopically normal-appearing peritumoral area^5^. On top of this, GBMs evade and suppress the immune system in the tumor microenvironment^6^.

Combination therapy, (i.e a treatment that combines at least two therapeutic agents) is often more efficient than a single modality treatment^7^. In this study, we treated tumor bearing rats with an approach that combines inducing significant tumor necrosis with delivery of chemotherapy to the tumor and infiltrating zone. Inducing necrosis within the tumor mass may be beneficial since GBM cells are often resistant to apoptotic stimuli but are more susceptible for cellular necrosis^8^. Furthermore, inducing necrosis was found to induce a tumor specific immune response^9,10^. In order to successfully treat the infiltrating cells with high chemotherapy doses, the BBB should be disrupted in areas surrounding the tumor mass. Since GBMs are vascularized tumors^3^, it is possible to use the tumor’s vasculature for drug delivery. Tissue necrosis within the tumor mass surrounded by BBB disruption can be achieved by electroporation (EP).

During EP external pulsed electrical fields are applied to cells or tissues, increasing the electrical potential across the cell membrane. The increased potential facilitates the creation of nanoscale aqueous pores in the lipid bi-layer, increasing the permeability of the cell membrane to macromolecules^11,12^. If the effect is transient and the pores are resealed it is termed reversible EP (RE), if EP causes cell death it is termed irreversible EP (IRE)^13^. Several factors contribute to the difference in effect including the size of the cells, the type to the tissue and electrical pulses parameters: number of pulses, amplitude, frequency, duration, and pulses shape. For a specific set of parameters, the most important one is the local electric field^14^.

Both IRE and electrochemotherapy (ECT, RE combined with chemotherapy) are showing promise in treating solid tumors outside the CNS^15–20^. While IRE is a method for tumor ablation^21,22^, EP is used to increase the uptake of chemotherapy into tumor cells in ECT^15,16^. The most common drugs that are combined with RE are Bleomycin, and cis-diamminedichloroplatinum II (Cisplatin)^16^.

It was demonstrated by us and others that when EP is applied to the brain with specific treatment parameters, the BBB is reversibly disrupted^23–25^. IRE applied to canine brain tumors was found to be safe, with little adverse effects, and to significantly reduce tumor volume^22,26^.

The most common EP treatment protocols employ either RE or IRE using an array of 2–8 electrodes^26,27^. In order to avoid craniotomy and to achieve both tumor ablation surrounded with BBB disruption we have developed a unique setup for brain tumors. This setup is minimally invasive and employs only one intracranial needle, insolated except for an exposed tip (1 mm for rat experiments), which is placed within the tumor mass. The second electrode is a surface electrode pressed against the rat skin^24,28^. This setup produces an electric field distribution which is strongest near the electrode exposed tip and decays with the square of the distance. Therefore, the electric fields within the tumor induce IRE effects that gradually taper down to reversible EP and BBB disruption at the infiltrating zone. This technique was termed point-source EP^24^.

In the current study, glioma-bearing rats were treated with a combination of point-source EP and systemic chemotherapy - Cisplatin or Methotrexate (MTX). Cisplatin^29^ is a potent antitumor agents, used against a wide range of solid tumors. MTX is used for the treatment of CNS metastases and systemic lymphoma and showed significant antineoplastic effects when combined with local BBB disruption techniques in glioma-bearing rats^30^. Here we aimed at studying the efficacy of combining peripheral administration of therapeutic agents with point-source EP treatment in tumor-bearing rats.

## Results

Three sets of experiments were conducted using 120 male Lewis rats, 280–320 gr at the day of tumor inoculation. Additional 11 tumor bearing rats died during the first 24 hours. All rats died from the anesthesia. Three rats died immediately post anesthesia administration, 4 rats died during MRI and 4 rats didn’t wake up from the anesthesia and died during the night. On day 1 post treatment none of the rats exhibited signs of distress and no neurological deficits were observed.

### EP only treatment

Tumor-bearing rats were treated with either 90 or 180 pulses. The results were compared to the control rats. The results of statistical analysis clearly indicate that there was no significant difference in tumor growth rate between control group and EP only groups. The objective response rate (ORR) was zero for all rats in all groups. There was also no statistical difference between BBB disruption volumes of the 90 pulses group and the 180 pulses.

**Figure 1:**
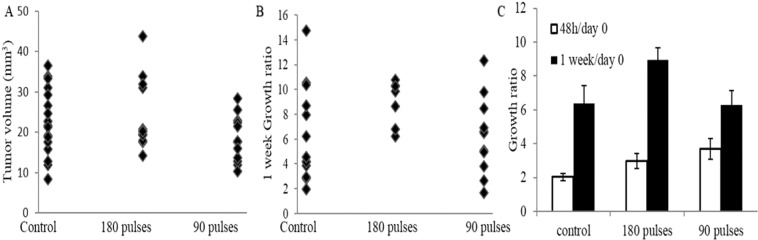
Tumor volumes and growth ratios of EP only experiment. (**A**) Tumor volumes for individual rats (**B**) 1-week growth rates, calculated as the ratio of tumor volume on follow up/tumor volume pre-treatment for individual rats one-week post treatment. (**C**) Mean tumors growth rates for 48 hours and 1 week.

Average tumor volumes and standard errors, calculated from contrast-enhanced T1-weighted MRIs (T1 MRIs) obtained prior to treatments were 22.7 ± 2, 18.4 ± 1.6 mm^3^ and 23.8 ± 2.4 mm^3^ for the control, 90 and 180 pulses groups, respectively, suggesting no significant differences in the baseline tumor volumes between the groups (ANOVA F(2,39) = 1.7, p < 0.19). Tumor volumes are presented in Fig. 1A.

No significant differences were found in the tumors growth rates between the groups at the two follow up time points, ANOVA F(2,33) = 2.7, p < 0.08, F(2,28) = 1.8, p < 0.18 for 48 hours and 1 week, respectively (Fig. 1B,C). A t test comparing the overall effects of EP (the 90 and 180 pulses groups were combined) with the control group also revealed no significant difference between the groups (P < 0.12), suggesting that applying EP treatment with the chosen parameters has no effect on brain tumors growth rates.

Average BBB disruption volumes calculated from contrast-enhanced T1 MRIs obtained 30 min post EP treatment were 129.7 ± 9.7 mm^3^ and 125.3 ± 8.9 mm^3^ for the 90 and 180 pulses group respectively. No significant differences in BBB disruption volume were found between the groups (t test, p < 0.74). No correlation was found between tumor volumes and BBB disruption volumes (r^2^ = 0.005, p < 0.87).

### EP with/without MTX

**Figure 2:**
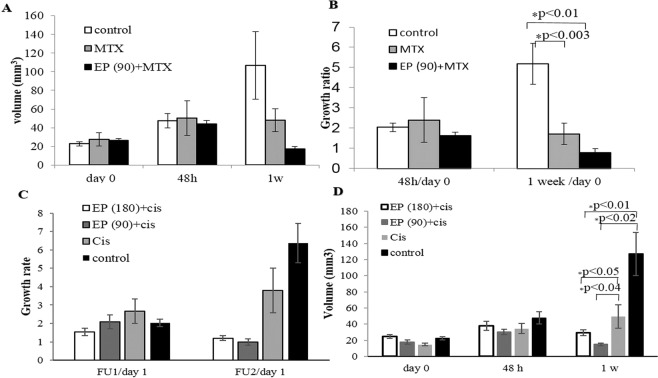
Mean tumor volumes and growth rates, calculated as the ratio of tumor volume on follow up/tumor volume pre-treatment. Error bars represent SE (**A**) MTX experiments-tumor volumes. (**B**) MTX experiments-growth ratios. (**C**) Cisplatin experiments-tumor volumes. (**D**) Cisplatin experiments-growth ratios.

Average baseline tumor volumes were 22.8 ± 2.3, 27.6 ± 7.3 mm^3^ and 25.2 ± 2.8 mm^3^ for the control, MTX and EP + MTX groups, respectively (Fig. 2A). There were no significant differences in the baseline tumor volumes between the groups (ANOVA F(2,23) = 0.4, p < 0.6).

Tumor growth ratio of rats treated with MTX, EP + MTX and controls were compared. The results of the statistical analysis demonstrate that EP-induced BBB disruption combined with MTX has the ability to slow tumor growth but has no significant additional benefit over treatment with MTX alone (Fig. 2B). This is in contrast to the fact that the objective response rate for the MTX group was 40% compared to 57% for the EP + MTX group.

ANOVA was used to examine whether there was a difference in tumors growth rates 48 hours post treatment. Since Levene’s F test revealed that the homogeneity of variance assumption was not met (F(2,23) = 4.87, p < 0.017), Welch correction was applied. The test revealed no significant differences in tumors growth rates, Welch’s F(2, 10.6) = 0.66, p < 0.53, indicating that 48 hours post treatment no treatment effects were found.

ANOVA was used to examine the differences in tumors growth rates 1 week post treatment. Since Levene’s F test revealed that the homogeneity of variance assumption was not met (Levene’s F(2,32) = 5.32, p < 0.016), Welch correction was applied.

The test revealed a statistically significant main effect: Welch’s F(2, 9.11) = 7.47, p < 0.012, ω^2^ = 0.35. These results indicated that approximately 35% of the total variation in the tumors growth rates were attributable to differences in the treatments.

Results of Games-Howell procedure indicated that the tumors growth rates in the combined EP + MTX group (1.02 ± 0.77) were significantly lower (p < 0.01) than the growth rates in the control group (5.2 ± 1.0 mm^3^), but there was no significant difference in tumors growth rates compared to the MTX only treatment group (1.7 ± 0.5 mm^3^). Therefore, the results showed that MTX alone was also effective in slowing tumors growth compared to control (p < 0.003, Fig. 2B).

### EP with/without Cisplatin

Tumor growth ratios of rats treated with EP + Cisplatin (90/180 pulses), Cisplatin and controls were compared. The results of this study suggest that EP + Cisplatin can delay tumors growth whereas Cisplatin alone has no effect on tumor growth compared to control. This is further strengthen by the ORR of the EP + Cisplatin groups which was 57% and 33% for the 90 and 180 pulses groups respectively, compared to 13% in the Cisplatin group and 0% in the control group.

A comparison between EP (90 pulses) + Cisplatin and EP (180 pulses) + Cisplatin demonstrated that average tumor volumes, calculated from contrast-enhanced T1 MRIs obtained before treatments, were 17.96 ± 2.7 mm^3^ and 24.6 ± 2.5 mm^3^ for the 90 and 180 pulses group, respectively. No significant differences in tumor volumes were found between the groups (t test, p < 0.07). Average BBB disruption volumes calculated from contrast-enhanced T1 MRIs obtained 30 min post EP treatment were 123.8 ± 5.0 mm^3^ and 137.0 ± 7.8 mm^3^ for the 90 and 180 pulses groups, respectively. No significant differences in EP-induced BBB disruption volumes were found between the groups (t test, p < 0.16).

ANOVA was used to examine whether there were differences in tumors growth rates 48 hours post treatment between the two EP + Cisplatin groups, Cisplatin alone and control groups. However, Levene’s F test for homogeneity of variance was significant (Levene’s F(3,41) = 3.9, p < 0.051). As such, the Welch correction was applied. The test revealed no significant differences in tumors growth rates, Welch’s F (2, 21.37) = 1.11, p > 0.19. These results indicate that 48 hours post treatment there were no treatment effects (Fig. 2C,D).

ANOVA was used to examine the differences in tumors growth rates 1 week post treatment. Since Levene’s F test was significant (Levene’s F(3,39) = 6.65, p < 0.001),Welch correction was applied. The test revealed a statistically significant main effect, Welch’s F(3, 39) = 64.7 p < 0.0003, ω^2^ = 0.38. These results show that approximately 38% of the total variation in tumors growth rates were attributable to differences in treatment.

Post hoc comparisons, using the Games-Howell procedure were conducted to determine which treatments differed significantly. These results showed that tumors growth rates in both combined EP + Cisplatin groups (0.98 ± 0.2 and 1.2 ± 0.1 for the 90 pulses and 180 pulses groups respectively) were lower than the growth rates in the control group (6.4 ± 1.0). P values and effect sizes were p < 0.02, 1.9 and p < 0.01, 2 for the 90 pulses and 180 pulses groups respectively. Tumor growth rates were also significantly lower compared to the Cisplatin group (3.8 ± 1.0), P values and effect sizes were p < 0.04, 1.9 and p < 0.05, 2 for the 90 pulses and 180 pulses groups respectively (Fig. 2D). Additionally, there were no significant differences between tumors growth rates in the control and Cisplatin groups (p < 0.3, Fig. 2D). Effect sizes for the significant effects were 1.9, 2,0.9,1 for 180 pulses/control, 90 pulses/control, 180 pulses/Cisplatin and 90 pulses/Cisplatin, respectively

No significant differences were found between the tumor volumes pre-treatment and at 1 week post treatment in both EP + Cisplatin groups (paired t test, p < 0.85).

**Figure 3:**
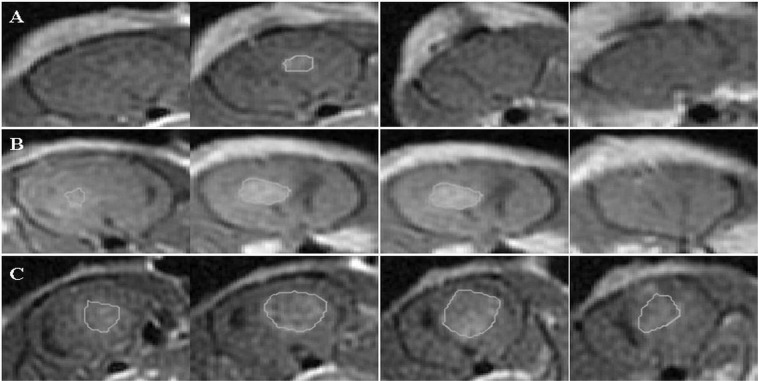
Contrast-enhanced T1-weighted MRI with regions of intrests of a rat from the control group. Four adjacent slices are shown at each time point, with the region of interest used to calculate the tumor volumes. Slice thickness is 2 mm. (**A**) tumor volume before treatment. (**B**) tumor volume 48 hours post treatment. (**C**) tumor volume 1 week post treatment.

**Figure 4:**
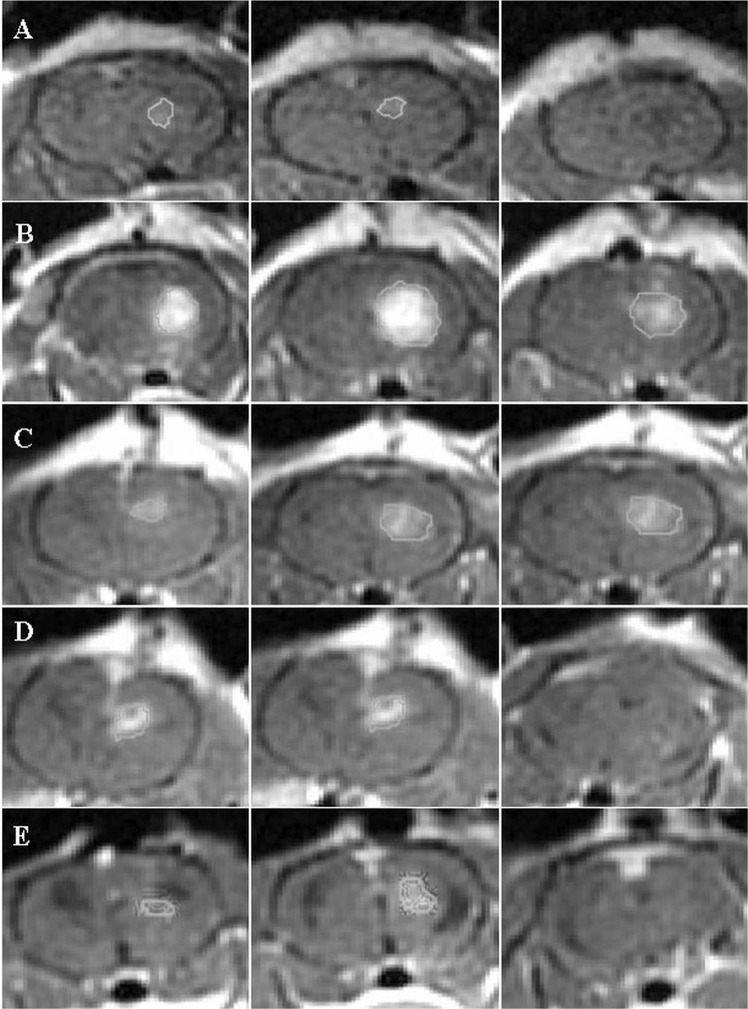
Contrast-enhanced T1-weighted MRI with regions of intrests of a rat treated with EP (90 pulses) + Cisplatin. Three adjacent slices are shown at each time point, with the region of interest used to calculate the tumor volumes. Slice thickness is 2 mm. (**A**) tumor volume before treatment. (**B**) BBB disruption 30 min post EP. (**C**) tumor volume 48 hours post treatment. (**D**) tumor volume 1 week post treatment. (**E**) tumor volume 2 weeks post treatment.

Examples of MRIs used to calculate tumor volumes at the different time points can be seen in Fig. 3 (control) and Fig. 4 (EP 90 pulses + Cisplatin).

### Survival analysis

**Figure 5:**
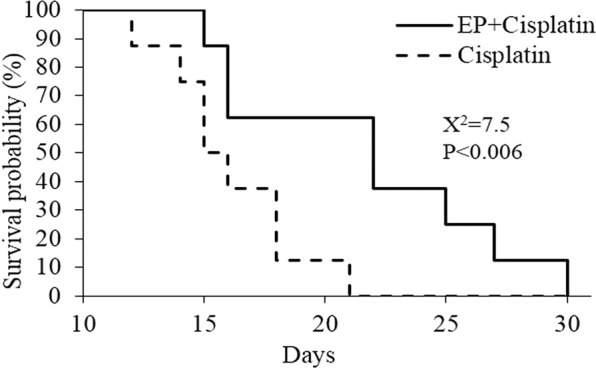
Kaplan Meier survival curve of rats treated with a combination of EP + Cisplatin and Cisplatin alone. Log Rank test revealed a significant difference between the groups, Χ^2^ = 7.54, p < 0.006.

A Log Rank test was performed to determine whether there was a significant difference in survival between the two groups of treatments: EP (180 pulses) + Cisplatin and Cisplatin. The mean and median survival for the two groups were 21.63 ± 1.97, 22 and 15.75 ± 0.96, 15 days for the EP + Cisplatin and Cisplatin alone respectively. Log Rank test revealed a significant difference between the groups, Χ^2^ = 7.54, p < 0.006, suggesting that combined EP + Cisplatin prolonged survival. The Kaplan-Meier curve is shown in Fig. 5.

### Histological analysis

H&E staining analysis of tumor-bearing brains extracted immediately post treatment showed clear differences between Cisplatin-treated or control brains to brains of rats treated with EP.

No cell death was apparent in the EP-treated brains, although some of the cells were preserved while others showed shape changes reflected in more spindling. Granulocytes and edema were visible. Hemorrhages were visible as well in the treated brains. The hemorrhages were limited to the tumors regions and were not spread to normal surrounding tissue. The hemorrhages were also visible in gradient-echo (GE) MRI. Control and Cisplatin-treated brains showed only viable cells with no signs of hemorrhages or spindling cells with less edema.

In brains of rats treated with EP + Cisplatin and extracted 48 hours post treatment it appears that the tumors were heavily bleeding. Many spindling cells were apparent. The tumors were undergoing microcystic changes, part of the cells were viable and others appeared stressed, with areas of decellularization depicted as rarified areas and extensive edema.

**Figure 6:**
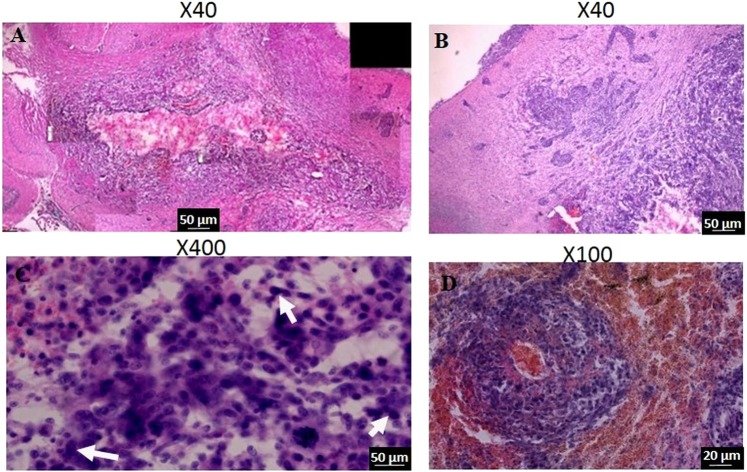
Cisplatin- induced effects 1 week post treatment, as determined by H&E staining. (**A**) Highly infiltrative tumor with necrotic core. The image was constructed using 8 images captured at the same conditions. (**B**) infiltration into the meninges. (**C**) viable tumor with many mitotic cells (arrows). (**D**) spontaneous bleeding in the tumor.

**Figure 7:**
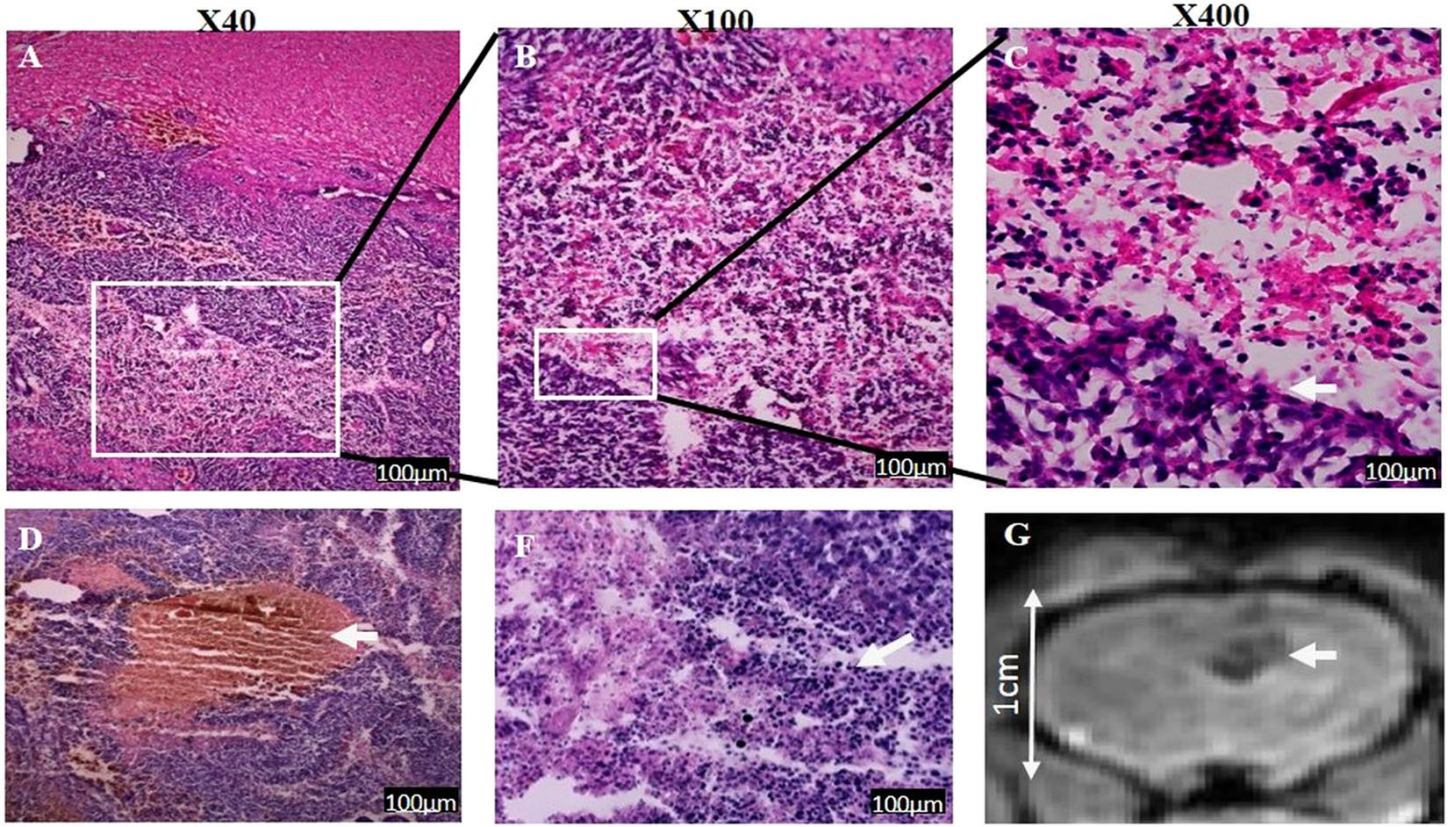
EP + Cisplatin- induced effects 1 week post treatment, as determined by H&E staining and MRI. (**A**) EP-induced necrosis in the tumor mass (**B**) larger magnification of necrosis (X100). The necrotic area contained mainly cell debris. (**C**) a demarcated border between necrosis and tumor (**D**) bleeding in the tumor mass. (**F**) infiltration of macrophages and lymphocytes into the tumor, mainly near the EP-induced necrosis border. (**G**) GE MRI showing bleeding in the treatment location.

In brains of rats treated with EP and extracted 1 week post treatment, necrotic areas were visible in the tumor core. Surrounding the necrosis the tumor seemed viable with large numbers of mitoses. Pseudo palisading necrosis was visible in several locations in the recovering tumors. Bleeding was still visible (Fig. 6). In brains extracted from rats treated with Cisplatin (Fig. 7) smaller bleedings were also visible. There was significant tumor infiltration into blood vessels and infiltration through the Virchow-robins space into the meninges. In contrast, No infiltration into the meninges was visible in any of the brains treated with EP + Cisplatin. In general, tumors treated with Cisplatin alone appeared to be more infiltrative and viable.

In brains treated with EP alone and extracted 7 days post treatment, infiltration into the tissues and meninges was also visible, and bleeding was present in the tumor mass. The cells were rearranged around the EP-induced necrosis. Cells adjacent to the rarified area did not recover, but were viable further away from the necrotic region.

Overall, tumors treated with EP + Cisplatin were smaller, seemed less infiltrative and appeared less viable than tumors treated with Cisplatin alone or EP alone. The effect of the treatment was visible as early as 48 hours post treatment, although MRI showed continuous tumor growth at this time point. One week post treatment the tumors were smaller but seemed to recover from the effect of the treatment. This is in accordance with MRIs obtained two and three weeks post treatment, showing a general increase in tumors volumes (data not shown). Bleeding was observed in most of the EP-treated tumors, either along the electrode path or at the tip, even though it did not occur in the naïve brains using the same protocol^24^. Although tumors treated with EP alone showed EP-induced necrosis in the tumor core, the tumors cells seemed more viable than with the combined treatment and the tumors were larger and more infiltrative.

## Methods

### Animals

The study was approved by and performed in accordance with the guidelines of The Animal Care and Use Committee of Sheba Medical Center and according to ARRIVE guidelines.

Three sets of experiments were conducted using 128 male Lewis rats, 280–320 gr at the day of tumor inoculation. Rats that died within 24 hours from the treatment day were excluded from the analysis. Only rats with initial tumor volumes between 7–40 mm^3^ were included in the analysis.

All rats were maintained on a 12:12-h light-dark cycle and provided food and water ad libitum. Rats were monitored for weight loss and signs of discomfort/distress on a daily basis. Rats were administered dipyrone through the drinking water for 5 days post treatment. Rats exhibiting a weight loss of 20% or more total body weight or high levels of discomfort and stress (inability to rise or move about the cage) were euthanized.

### Experimental outline

Experiment #1: The goal of this experiment was to evaluate the effect of combined point-source EP and chemotherapy on tumor growth rates. Rats were treated using two different EP protocols, 90 of 180 pulses, and two types of chemotherapy, intra-venous (IV) Cisplatin (4 mg/kg)^31,32^ or intra-peritoneal MTX (6 mg/kg)^30^. A total of 87 rats were included in the final analysis. Rats were scanned by MRI prior to treatment to obtain baseline tumor volumes and divide into treatment groups. Follow-up MRIs were performed 48 hours and 7 days post treatment. The different treatment groups are listed in Table 1. The study was conducted in 6 separate experiments.

**Table 1:** Experiment #1 - treatment groups and the number of rats in each group.

Treatment	# rats
IV Cisplatin (4 mg/kg)	15
EP (180 pulses, 600 V, 50µs pulses at 1 Hz) + IV Cisplatin (4 mg/kg)	9
EP (90 pulses, 600 V, 50µs pulses at 1 Hz) + IV Cisplatin (4 mg/kg)	9
Control	17
EP (180 pulses, 600 V, 50µs pulses at 1 Hz)	13
EP (90 pulses, 600 V, 50µs pulses at 1 Hz)	13
IP MTX (6 mg/kg)	6
EP (90 pulses, 600 V, 50µs pulses at 1 Hz) + IP MTX	7

Experiment #2: The goal of this experiment was to evaluate the effect of treatment on survival. 16 rats were scanned by MRI prior to treatment to obtain baseline tumor volumes and divide into 2 treatment groups, EP + Cisplatin (n = 8) and Cisplatin (n = 8). EP protocol included 180 pulses 600 V, 50 µs pulses at 1 Hz. There was no follow-up by MRI in order to avoid death due to repeated anesthesia that might compromise the survival analysis.

Experiment #3: The goal of this experiment was to study histological response to the treatment. 25 rats were scanned by MRI prior to treatment to obtain baseline tumor volumes and divide into 5 treatment groups: EP, EP + Cisplatin/MTX, and chemotherapy alone. Following MRI, rats’ brains were extracted either immediately post treatment, 48 hours post treatment or 1 week post treatment and were subjected to histological analysis.

### Cell culture preparation

CNS1 rat glioma cells (orthotropic rat glioma model developed in inbred Lewis rats)^33^ were maintained at 37 ^o^C and 5% CO_2_ in Dulbecco’s Modified Eagle Medium (DMEM) enriched with 10% fetal calf serum and penicillin/streptomycin 1% and were subcultured twice a week. For tumor inoculation, a pellet of 1 × 10^8^ cells suspended in 2 mL Phosphate buffered saline (PBS) was prepared.

### Tumor inoculation

Rats were anesthetized by intra muscular injections of 0.75 ml/kg Ketamine and 1.3 ml/kg Xylazine. A midline scalp incision was made to identify the bregma. A 1 mm burr hole was made in the right region of the skull, 3 mm anterior and 2 mm lateral to the bregma. A 32 G needle attached to a 1 ml syringe containing the CNS1 pellet was placed stereotactically 5.5 mm deep into the striatum. The cells were infused at 2 µl/min for 5 min to a total of 5 × 10^5^ cells per rat. After the termination of the inoculation, the burr hole was sealed with bone wax and the incision was sutured.

### Experiment procedure

On day 5 after tumor inoculation, rats were scanned by MRI in order to evaluate the baseline tumor size and location. Gd-based contrast agent was injected IP (Gd-DOTA, 0.015 mmol/kg, Dotarem, Guerbert) 20 min prior to MRI. Rats were then divided into the 6 treatment groups based on their tumor volumes so that the tumor volume distributions were similar for all groups.

Following the baseline MRI the rats remained under full anesthesia and were treated approximately 55 min post MRI to enable clearance of the contrast agent. The burr hole used for tumor inoculation was re-opened. A custom designed needle electrode (30 gauge, silver plated copper electrode), insulated except for a 1 mm exposed tip, was placed stereotactically into the tumor mass. A flat 3 × 5 cm ground electrode was pressed against the rat chest after applying conducting gel for improved electric coupling. The electrodes were then connected to a pulse generator (BTX ECM 830, Harvard Apparatus, Holliston, MA). Gd-DOTA, 0.015 mmol/kg, was injected IP 1 min prior to treatment. For rats treated with EP + Cisplatin, Cisplatin (Abiplatin 1 mg/ml solution for injection, Pharmachemie BV Holland) was injected IV at a dose of 4 mg/Kg with no additional dilution 1 min prior to treatment. MTX (Abitrexate 25 mg/ml solution for injection, Pharmachemie BV Holland) was injected IP 20 min prior to treatment at a dose of 6 mg/kg (dilution 1:3 with saline). EP treatment consisted of 50 µs monopolar electric pulses at a frequency of 1 Hz and treatment voltage of 600 V. Either 90 or 180 pulses were applied.

Thirty min post treatment the rats underwent a second MRI to evaluate the treatment effects. Rats in the control and Cisplatin groups were subjected to the same protocol without applying the electrical pulses.

Rats treated with MTX, were also administered 6 mg/kg MTX on days 2 and 4. In order to avoid MTX-induced systemic toxicity, 8 mg/kg of Leucovorin (LV, 10 mg/ml solution for injection Pharmachemie BV Holland), a folinic acid that competes for active transport with MTX, was administered on days 1,3,5 diluted 1:3 with saline .

### Imaging and image analysis

Contrast-enhanced T1 MRI was used for evaluating tumor volumes and EP-induced BBB disruption. Controls were also scanned to ensure the effect is solely from the EP treatment and not from the added contrast agent. Tumor growth rates were calculated from contrast-enhanced T1 MRIs obtained 48 hours and up to 2 weeks post treatment. Treatment effects, including edema and bleeding, were evaluated by T2-weighted MRI (T2 MRI) and GE MRI.

EP-induced BBB disruption and tumor volumes (in mm^3^) were calculated by plotting regions of interest (ROIs) over the entire enhancing region in each slice (excluding the ventricles). Delineated ROIs are shown in Figs. 2 and 3. The number of pixels in the ROIs was then counted and multiplied by the volume of a single pixel. Slice thickness was 2 mm and in-slice pixel dimensions were 0.3 × 0.3 mm.

### Histological analysis

Twenty five rats brains treated with either EP, Cispatin or EP + Cispatin were extracted post MRIs at different time points. EP protocol included 90 pulses at 600 V, 50 μs pulse duration at 1 Hz. As the results of the MRI and efficacy study revealed no significant difference between 90 and 180 pulses, the shorter treatment was preferred. Brain tissue was fixed at 4% neutral buffered PFA for at least 48 hours and then cryoprotected in PBS containing 15% sucrose solution at 4 °C for 24 hours. Brains were then frozen in dry ice and kept at −70 °C. The tissue was embedded in OCT compound and then serial sectioned in the coronal plane (20 μm). Sections were placed onto glass slides (Superfrost plus slides, Thermo scientific) and stored in a sealed slide box at −70 °C.

### H&E staining

After fixation in 4% neutral buffered paraformaldehyde for 1 min, sections were washed in running tap water X3, stained with Mayers Hematoxylin for 2 min and washed again in running tap water X3. Sections were dipped in Ammonium Hydroxide 32% (in diluted water) for 0.5 min, washed in tap water X3 and stained with Eosin phloxin for 0.5 min. Sections were washed again in tap water X3 and dehydrated through 4 changes of 100% ethanol (0.5 min each). Slides were then cover slipped. Slides were examined under a light microscope (Nikon 50i).

### Numerical simulation

A numerical simulation was performed using COMSOL Multiphysics 4.3 to calculate the electric fields distribution within the tumor and the infiltrative zone as previously described^24,28^.

In short: The rat head and chest were modeled as a 20 × 20 × 18 mm ellipsoid with an initial conductivity of 0.258 S/m. The electric field was described by the Laplace equation for electric potential distribution in a volume conductor:∇ (*σ*(E)∇*φ*) = 0 where σ is the electric conductivity of the tissue, *E* is the applied electric field and *φ* is the potential. The *σ(E)* dependence of brain tissue was described by a sigmoid curve with a transition zone between 500 V/cm and 700 V/cm, in which the conductivity changes from 0.258 S/m to 0.516 S/m as described by Ivorra a *et al*.^34^. Dirichlet boundary condition was applied to the surface of the electrode: φ = φ_0_ and to the ground: φ = 0 where *φ*_0_ is the applied potential on the intracranial electrode. The boundaries where the analyzed domain was not in contact with an electrode were treated as electrically isolative and Neumann boundary condition was set to zero on the outer border of the model: $\frac{\partial \varphi }{\partial n}=0$, where *n* denotes the normal to the boundary. A spherical 1.7 mm radius tumor (to match the average tumor radius of the rats treated with EP) was added to the model. The conductivity of the tumor was set as 0.516 S/m (brain conductivity x2) and decreased with the distance for additional 4 mm in a sigmoidal manner down to 0.258 S/m in order to incorporate tumor heterogeneity and the infiltrative zone in the model.

**Figure 8:**
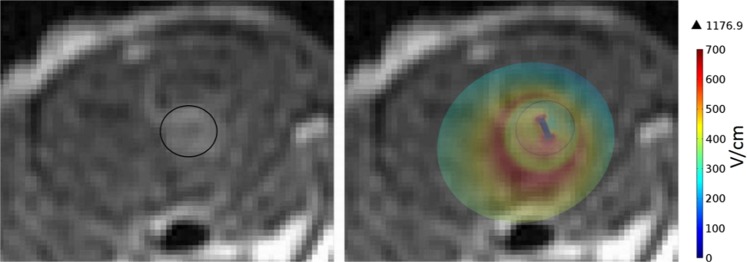
Electric field distribution in the tumor and infiltrative zone calculated using COMSOL multiphysics and fitted on MRI of rat brain with a tumor of 1.7 mm radius.

Figure 8 shows electric filed distribution. Although the electric field isn’t uniform, the entire tumor and infiltrating zone experience electric field above 500 V/cm which was found to be the threshold for EP-induced BBB disruption by us and others. It is also clear from the results that IRE threshold is only achieved in small portion of the tumor.

### Statistical analysis

Power analysis was performed to calculate sample size for the efficacy study. Table 2 lists the sample sizes required for specified effect sizes, in units of Cohen’s d. Group sizes were based on the assumption that effect size will be large (above 0.8).

**Table 2:** Power analysis for sample size calculation, based on effect size in units of Cohen’s d.

EFFECT SIZE	0.8	0.5
POWER	Rats per group	
0.8	6	14
0.85	7	16
0.9	8	18
0.95	10	22

Growth rates were calculated as the ratio between the tumor volume 48 hours or 1 week post treatment and the tumor volume on the treatment day.

Three different analyses were conducted:

A one-way analysis of variance (ANOVA) was conducted to compare the effects of EP alone (90 or 180 pulses) with no treatment (control) on tumor growth.

ANOVA was conducted to compare the effects of Cisplatin, EP (90 pulses) + Cisplatin, EP (180 pulses) + Cisplatin and no treatment on tumor growth. Separate ANOVA compared the effects of MTX, EP + MTX and no treatment on tumor growth.

Homogeneity of variance was analyzed using Leven’s test and if the test was significant, Welch correction was applied. Post hoc analysis included Games-Howell test.

ORR was determined as the change in tumor volume at follow-up compared to tumor volume at the treatment day.

### Ethics approval

The study was approved by and performed in accordance with the guidelines of The Animal Care and Use Committee of Sheba Medical Center. Consent for publication: Not applicable

## Discussion

Primary brain tumors are extremely difficult to treat. The tumors are often resistant to apoptotic stimuli, are infiltrative by nature and the BBB prevents penetration of therapeutic drug doses into the tumor mass and even more into the infiltrative zone^4^. The optimal treatment should thus provide focal damage in the tumor mass in parallel to BBB disruption in the infiltrating zone, enabling localized high dose chemotherapy. Both ECT^27^ and IRE, as mentioned in the Introduction, although very promising, provide focal solutions to the tumor mass but do not address the issue of infiltrating cells which are a major cause for tumor recurrence^5^.

The mode of treatment presented here, point-source EP, provides in parallel focal destruction of the tumor mass, and efficient treatment of the infiltrating tumor cells. The unique electric fields distribution that is produced by the point-source setup creates high electrical fields within the tumor core, inducing IRE, and lower electric fields at larger distances from the electrode, inducing BBB disruption in the infiltrative zone^24^.

The presented experiments were designed to evaluate the efficacy of a combined treatment of point source EP with systemic chemotherapy.

The MRIs obtained immediately post EP indicated that BBB disruption was always larger than the tumor volumes thus the BBB was disrupted in the infiltrating zone and not only in the tumor. Nevertheless, our results demonstrated that point-source EP as a single modality had no benefit in delaying tumor growth. Tumor volumes continued to increase as well as the infiltration of tumor cells. Histological analysis indicated that the damage induced by IRE was smaller than the tumor, enabling the residual tumor to continue growing while the infiltrative zone remained untreated. As increasing the number of pulses or pulse amplitude may increase the damage volume, it is possible that choosing different treatment parameters would yield different results. On the other hand, tumors that were treated with EP + Cisplatin clearly demonstrated delayed tumor growth.

Although Bleomycin is the most common drug in combination with EP, it does not penetrate the cells membranes without EP. here we chose to use MTX and Cisplatin since it is possible that the endothelium may be reversibly electroporated in lower electrical fields than tumor cells. Sersa *et al*.^35^ also demonstrated that tumor blood vessels are exposed to ∼40% higher electric field than the surrounding tumor cells, and therefore easily electroporated. If so, it may be possible to obtain larger affected volumes in the infiltrative zone if the drugs penetrate the cells using different (other than EP) mechanisms.

MTX is not considered highly cytotoxic and enters the cells in two ways: active transport, and diffusion in concentrations greater than 20 µM. MTX can penetrate over the BBB to some extent, especially at high dosage^36^, and is used for the treatment of CNS metastases and CNS lymphoma.

In our experiment, MTX treatment alone was found to be effective in slowing tumor growth with no additional significant benefit to EP. Nevertheless, the ORR was higher in the EP + MTX group (57% compared to 40%) which may suggest some marginal benefit of the combination with EP. This result is in contradiction with the results of Isobe *et al*.^37^ who showed that a single treatment of reversible EP with systemic MTX decreased substantially the size of osteosarcoma tumors in mice compare to controls. Since in our experiment MTX was efficient in slowing tumor growth by itself, one explanation could be that sufficient MTX dosage passed the BBB without EP (which is already slightly disrupted in the tumor mass) with no additional benefit for larger BBB disruption.

Our results indicate that EP + Cisplatin can slow tumor growth (increase ORR) and increase survival. These results are in accordance with other ECT experiments conducted on tumors outside the CNS. Cisplatin enters the cells via two mechanisms. A fraction of the Cisplatin molecules enter the cells by diffusion, whereas another fraction enters through a facilitated transport^38^. Previous *in vitro* and *in vivo* experiments showed that EP increases Cisplatin toxicity by a factor of 2.3–80^19,31,39,40^ depending on the experimental conditions.

One limitation of using Cisplatin is that it may cause severe brain toxicity. When the BBB is disrupted as in the combined treatment group, large portions of the brain are exposed to the drug, causing increased brain toxicity. We found that rats in the EP + Cisplatin group suffered from immediate weight loss, potphyrin discharge around the eyes and nose, and some rats showed transient partial paralysis of the lower limbs. These side effects were also observed in rats treated with Cisplatin alone, but to a lesser extent.

The treatment effects were only visible by MRI 1 week post treatment. Reasons for this phenomenon may include the limitation of MRI as a method that evaluates lesions volumes. As the MRI depicts the BBB disruption volume caused by the fenestrated and damaged blood vessels of the tumor, 48 hours might not be enough for the destruction of these vessels although tumor cells lysis has already occurred. Another possible reason is that the BBB remains open for over 48 hours and that some treatment effects take longer than this time period.

Even though Cisplatin is a highly potent drug, it is rarely used systemically for the treatment of brain tumors due to insufficient BBB penetration. Direct drug administration, although can potentially increase local drug concentration without increasing systemic concentration, is severely restricted by the limited diffusion of drug through the tissue, in the order of a few mm. Furthermore, platinum derivatives administered directly were found to induce seizures, encephalopathy, stroke, ataxia and/or myelopathy^41,42^.

Agerholm-Larsen *et al*. have shown that ECT with intra-tumoral injection of Bleomycin induced regression and elimination of tumor in 9 of 13 treated rats over 2–3 weeks^27^. These results are encouraging although the baseline average tumor volume in this experiment was only 7 mm^3^, significantly smaller than in our experiments, and the authors stated that larger tumors had lower success rate. One possibility is that the lower success rate observed in our experiment was due to the larger tumor volumes, although it is also possible that a single treatment is not enough to eliminate these aggressive tumors.

### Limitations of the treatment

Both GE MRI and histological analysis demonstrated hemorrhages occurring in the tumors after EP. In most cases the hemorrhages were depicted along the path of the electrode which might suggest that they were caused by electrode insertion or moving during the treatment. But in some cases bleeding was depicted only at the treatment location, i.e the tumor core. This finding is in contrast to our previous experiments in naïve rats which showed no sign of hemorrhages^24^ and in contrast to the assumed mechanism of EP which spares blood vessels matrix^43^.

One possible explanation may be related to technical limitations of our model. The treatment causes muscle contraction. The movement can cause electrode movement in the brain, resulting in bleeding. This can be solved by administrating muscle relaxants. Since muscle relaxants can depress breathing, ventilation is needed, which was not available in our experimental setup.

Another explanation may be that tumor vasculature is more sensitive to EP-induced damage than normal blood vessels^44^. As the tumor vessels are damaged and fenestrated, they might not hold the pressure of the vasoconstriction occurring after EP and although blood flow is significantly reduced following EP^45^, the elevated pressure might be high enough to cause blood vessels rapture^35^.

Another limitation is the continued tumor growth after 2 weeks. Since the treatment was not sufficient to eliminate the entire tumor mass, the tumor cells recovered and continued to grow. The treatment parameters were chosen based on the results of previous experiments^24,30^ conducted on naïve brain. Since the tumors are denser and more conductive than normal brain, it is possible that increasing the treatment voltage or the number of pulses would induce increased antineoplastic effects. Another option, as the treatment is rapid, is to prevent re-growth by repeated treatments.

## Conclusions

This study demonstrated the efficacy of applying point-source EP for the treatment of brain tumors. The treatment is minimal invasive and rapid. We showed that when applying EP using a single intracranial electrode and an external surface electrode it was possible to induce controlled irreversible damage within the tumor mass surrounded by a reversible electroporation zone with enhanced uptake of chemotherapy. These are included in a larger volume of BBB disruption covering the entire tumor volume and the surrounding infiltrative zone. Although complete elimination of the tumors was not achieved, increased survival and response rates as well as reduced tumor growth rates were found for rats treated with EP + Cisplatin compared with control and Cisplatin alone. The next step towards treating human patients should be to adapt the protocol and the setup for larger volumes. The treatment should consist of, as demonstrated here, non-thermal irreversible damage to the tumor core surrounded by large transient BBB disruption for efficient drug penetration.

## Data Availability

The datasets used and analyzed during the current study (tumor volumes, survival, histological analysis) are available from the corresponding author on reasonable request.
